# Medical Colleges and Medical Education in the South

**Published:** 1892-09

**Authors:** 


### Editorial.

### MEDICAL COLLEGES AND MEDICAL EDUCATION IN THE SOUTH.

Three of our Southern medical journals, *The Virginia Medical Monthly* of July, the *Atlanta Medical and Surgical Journal* of August, and the *Alabama Medical and Surgical Age* of August, contain editorials on this subject; the former, in all spirit of fairness, and the two latter in a *decided* spirit of unfairness. I say the two latter, but probably it would be better to say the *Atlanta Medical and Surgical Journal* alone, for the editorial in the *Alabama Medical and Surgical Age*, with the exception of a paragraph at the beginning, and one at the ending, is copied from the *Atlanta Medical and Surgical Journal*; and strange to say, both journals are *August* numbers. So, with the exception of the copied editorial and the credit it claims of having first published "the unique table given in his annual address of the medical colleges in this country, and the medical examining board of Alabama, by Dr. B. J. Baldwin," they can be put aside.

Now, before taking up the editorials for review of the other journals, we have a word to say in defense of the Southern colleges, and the work they have accomplished. For we know that medical education in the South is not at the low ebb the editorial in the *Atlanta Medical and Surgical Journal* would have us believe.

The Southern colleges have graduated men capable of doing as good, honest, conscientious work—as brilliant work—as any in this country. No part of the world has produced as many pioneers in the advancement of the medical sciences in proportion to their numbers as the South, whose achievements have done more to alleviate the sufferings of humanity than any others. It would be useless to cite illustrations, for they are known to all who are familiar with medical progress.

The older colleges of the South have progressed under great disadvantages; they were torn up and used for the reception of our wounded during the war. After this cloud had passed away, and the professors who were so fortunate as to survive their active service in the front had returned, they began to re-organize. Sadly the faculties met, and with sorrow, filled the vacancies caused by the death of those poor fellows who had given up their lives in the defense of that which they loved so well.

Immediately they went to work, and nobly and successfully did they strive to refit their dismantled schools; going into their own pockets for funds. For, as many of the older physicians can testify, they frequently graduated men who have since become distinguished, who, at that time, did not have money enough to pay their matriculation fees, taking the notes of the poor fellows rendered destitute by no fault of theirs except the love of their country, depending simply upon their honor for them to be redeemed, should they ever become able to do so. I am glad to say these honest, hard-working physicians have almost invariably redeemed their pledges, where they have not passed away with the ebbing tide before it was possible.

This action of the colleges was a necessity, for nearly all the schools had been closed during the whole period of the war. Nearly all the physicians enlisted in active service, and went to the front; but many, many of the poor fellows never returned. So that, in many vicinities, physicians were absolutely necessary.

And to-day, may it be said to their shame, many of the younger generation point to the noble institutions to whom the South owe so much, with a finger of scorn, and call them cheap colleges, on account of their action during that time.

Year by year, ever striving for higher things, have the faculties of these schools improved their buildings and extended their facilities for teaching, and may it be said to their credit, going into their own pocket for funds, and making their personal sacrifices without a murmur. And so far as my personal knowledge of the schools of this State, goes never, but with a single exception, and that of recent date, through one of the papers of this city, calling on the laity for assistance to complete and equip their school.

Now in the same journal, the Georgia medical schools are taxed for not requiring a three-years term. Some of the schools in this State, to my certain knowledge, offer to their students free tuition for the third term if they will take it, and I am glad to say that the number of those who are taking advantage of this offer are increasing each year.

During the last session of the Legislature of this State, there was a bill before it to enact a law requiring a three-years course of the students of the colleges of this State before they could apply for graduation.

All the colleges seemed to be in favor of it, and my understanding was that there was a tacit agreement that each one of the faculties of the different schools should aid the passage of this law. But, lo! and behold, just as the bill seemed to have every chance of passing by a large vote, the most prominent member of one of the medical schools of this city—a man of rather short but decidedly rotund figure, smooth-faced and the size of whose cheek-s is only equalled by the circumference of girth—appeared before the committee having charge of this bill, and in a speech of large volume and small substance argued against its passage. The bill did not pass. I suppose the committee agreed to kill the bill to stop the speech; for certainly neither the good looks or fascinating manners of this individual could possibly have had any effect.

Certainly the medical schools of the South have, in the most *positive* way, expressed their *very high appreciation* of the *ability* of at *least one school* of this *section* to *teach* the *very best* and *progressive medicine*, for I see that the Southern Medical College of this city has filled two of its most important chairs with recent graduates from the ATLANTA MEDICAL COLLEGE of this city, one being elected Professor of Physiology, and the other Professor of Chemistry and Lecturer on Pathology. So at *least one* of our colleges graduates students who will make satisfactory professors for other colleges, even colleges situated in the same city, where, of course, *one who did not know*, would *suppose competition existed.* Honor to those to whom honor is due.

We do not consider any statistics based upon successes or rejections before the examining boards (not board, for there is one for each county) of Alabama reliables. Our reasons for this opinion is not only derived from personal knowledge, but also upon the condemnation and censure as expressed by the Alabama State Medical Association in their transactions. We copy their own words from this volume, "It has been our disagreeable duty from time to time to be obliged to recommend the censure of our county boards of medical examiners. As a rule, these delinquent boards have recognized the justice of the censure visited upon them."

We should like to go into the methods of the Alabama examining boards more thoroughly, but for lack of space, defer it until another issue.

In closing this editorial, we simply cite the percentage of rejections as given by the editor of the *Virginia Medical Monthly*, who seems to have some pride in the rating of his own section, and who thinks the "Southern States medical colleges are more exacting as to the grade of requirements for medical graduation than are those of the West or North." In his article, the percentage of rejections is as follows :

Southern college graduates, 17.3 plus per cent.
Western college graduates, 18.2 plus per cent.
Northern college graduates, 35.3 plus per cent.

